# *In vitro* Activity of Pentamidine Alone and in Combination With Aminoglycosides, Tigecycline, Rifampicin, and Doripenem Against Clinical Strains of Carbapenemase-Producing and/or Colistin-Resistant Enterobacteriaceae

**DOI:** 10.3389/fcimb.2018.00363

**Published:** 2018-10-18

**Authors:** Tania Cebrero-Cangueiro, Rocío Álvarez-Marín, Gema Labrador-Herrera, Younes Smani, Elisa Cordero-Matía, Jerónimo Pachón, María Eugenia Pachón-Ibáñez

**Affiliations:** ^1^Clinical Unit of Infectious Diseases, Microbiology, and Preventive Medicine, University Hospital Virgen del Rocío, CSIC, University of Seville, Seville, Spain; ^2^Institute of Biomedicine of Seville, University Hospital Virgen del Rocío, CSIC, University of Seville, Seville, Spain; ^3^Department of Medicine, University of Seville, Seville, Spain

**Keywords:** Enterobacteriaceae, colistin-resistant, carbapenemase producers, pentamidine, *in vitro* activity

## Abstract

Enterobacteriaceae cause different types of community- and hospital-acquired infections. Moreover, the spread of multidrug-resistant Enterobacteriaceae is a public health problem and the World Health Organization pointed them among the pathogens in which the search of new antibiotics is critical. The objective of this study was to analyze the *in vitro* activity of pentamidine alone and in combination with gentamicin, tobramycin, amikacin, tigecycline, rifampicin, or doripenem against eight clinical strains of carbapenemase-producing and/or colistin-resistant Enterobacteriaceae: five carbapenemase-producing *Klebsiella pneumoniae*, one carbapenemase-producing *Escherichia coli*, and two colistin-resistant *Enterobacter cloacae*. MIC and MBC were determined following standard protocols. MIC results were interpreted for all the antibiotics according to the EUCAST breakpoints but for rifampicin in which the French FSM breakpoint was used. Bactericidal and synergistic activity of pentamidine alone and in combination with antibiotics at concentrations of 1xMIC was measured by time-kill curves. For one selected strain, *K. pneumoniae* OXA-48/CTX-M-15 time-kill curves were performed also at 1/2xMIC of pentamidine. All studies were performed in triplicate. Pentamidine MIC range was 200–800 μg/mL. The 50, 12.5, 62.5, 87.5, and 62.5% of the strains were susceptible to gentamicin, tobramycin, amikacin, tigecycline, and doripenem, respectively. Only the two *E. cloacae* strains were susceptible to rifampicin. Pentamidine alone at 1xMIC showed bactericidal activity against all strains, except for the *E. cloacae* 32 strain. The bactericidal activity of pentamidine alone was also observed in combination. The combinations of pentamidine were synergistic against *E. cloacae* 32 with amikacin and tobramycin at 24 h and with tigecycline at 8 h. Pentamidine plus rifampicin was the combination that showed synergistic activity against more strains (five out of eight). Pentamidine plus doripenem did not show synergy against any strain. At 1/2xMIC, pentamidine was synergistic with all the studied combinations against the K. pneumoniae OXA-48/CTX-M-15 strain. In summary, pentamidine alone and in combination shows *in vitro* activity against carbapenemase-producing and/or colistin-resistant Enterobacteriaceae. Pentamidine appears to be a promising option to treat infections caused by these pathogens.

## Introduction

Carbapenem-resistance in *Enterobacteriaceae* is a world health problem that has made the World Health Organization (WHO) to point it as a priority in the list of bacteria for which new antibiotics are urgently needed (World Health Organization, 2017). Moreover, these pathogens have spread globally in the past years, and are associated with carbapenemase production as the most important resistance mechanism (Cantón et al., 2012). Therapeutic options for infections caused by these kinds of pathogens are scarce and colistin is often the only remaining treatment option. However, the appearance of colistin-resistant strains has risen dramatically in the last decade due to antibiotic pressure in both human treatment and its use in agriculture (Kempf et al., 2016; Olaitan et al., 2016). In a recently published study (Hong et al., 2018), the *in vitro* activity of colistin was analyzed against 356 clinical strains of *Enterobacter* spp. from eight Korean hospitals, founding that 23.9 and 4.2% of *E. cloacae* and *E. aerogenes* strains, respectively, were resistant to colistin.

In this context, clinical experience on the most effective treatment for infections caused by these pathogens is still scarce (Akova et al., 2012). Currently, the majority of clinical studies conclude that the combined treatment with two or more antimicrobials is the better option in terms of increase the survival (Trecarichi and Tumbarello, 2017). Numerous studies, both *in vitro* and *in vivo*, have tested the efficacy of antimicrobials combinations against these kinds of pathogens (Pachón-Ibáñez et al., 2018; Wang et al., 2018). Nevertheless, the best combination depends on the susceptibility pattern of the strains and there is no one combination that we could qualify as optimal.

Therefore, the increase in the rates of antimicrobial resistance, the difficulty to find an optimal and effective treatment for infections caused by these pathogens, and the lack of the development of new families of antimicrobials by the pharmaceutical industry (Spellberg and Rex, 2013), make urgent the search for new approaches to combat the problem caused by multi-resistant strains of Gram-negative bacilli (GNB) and, specifically, by carbapenemase-producing and/or colistin-resistant Enterobacteriaceae.

As a new treatment strategy, the repurposing of drugs for the treatment of infections caused by these kinds of pathogens seems especially interesting (Younis et al., 2015). This new approach reduces the time, cost, and risk associated with the development of antimicrobial molecules *de novo*. In addition, its effectiveness has been demonstrated in different medical areas, such as infectious diseases (Debnath et al., 2012). Despite of that several drugs have been recycled for other clinical indications; none has been used for the treatment of bacterial infections.

Pentamidine (in the form of isethionate) is an antiprotozoal agent effective in trypanosomiasis, leishmaniasis, and some fungal infections (Nguewa et al., 2005). To our knowledge, pentamidine has never been used in clinic as antimicrobial agent. Nevertheless, in a recent study Stokes et al. found that pentamidine is able to disturb the outer membrane of GNB, due to the interaction with membrane lipopolysaccharides (Stokes et al., 2017). Moreover, they concluded that pentamidine in combination with antimicrobials typically used for Gram-positive cocci had synergistic activity *in vitro* against different GNB and in a mice sepsis model by *Acinetobacter baumannii*.

The aim of this study was to evaluate *in vitro* the activity of pentamidine alone and in combination with different antimicrobials against clinical strains of carbapenemase-producing and/or colistin-resistant Enterobacteriaceae.

## Materials and methods

### Bacterial strains

Eight clinical strains of carbapenemase-producing and/or colistin-resistant Enterobacteriaceae were studied: (1) five strains of carbapenemase-producing *Klebsiella pneumoniae*: Kp07, a VIM-1 ST 1603 clone producer from Spain (Miró et al., 2013); Kp21, co-producing VIM-1 and AmpC type beta-lactamase DHA-1 ST 11 clone from Spain (Miró et al., 2013); Kp28, co-producing OXA-48 ST11 clone and the extended spectrum beta-lactamase (ESBL) CTX-M-15 from Spain (Oteo et al., 2015); a Kp29, co-producing KPC-3 ST512 clone with the extended spectrum beta-lactamases TEM-1 and SHV-11 from Spain (López-Cerero et al., 2014); Kp1, a NDM-1 producer from Kenya; (2) Ec271, a *Escherichia coli* NDM-1 producer from Australia Docobo-Pérez et al., 2012; (3) two strains of *Enterobacter* spp. from Spain, *E. cloacae* 32 and *E. cloacae* 297, both resistant to colistin. Identification of these isolates was confirmed by a Microflex LT-MALDI Biotyper mass spectrometer (Ruiz-Aragón et al., 2018) (Bruker Daltonics GmbH, Bremen, Germany). The presence of carbapenemase genes, and genes coding for other beta-lactamases was confirmed by PCR and sequencing as described previously.

### Drugs

All the drugs tested were used as standard laboratory powders (Sigma-Aldrich, Madrid, Spain): pentamidine, aminoglycosides (gentamicin, tobramycin, and amikacin), tigecycline, rifampicin, and doripenem.

### Antimicrobial susceptibility testing

The minimal inhibitory concentration (MIC) and minimal bactericidal concentration (MBC) values were tested. MICs of antibiotics were determined by broth microdilution as recommended by the (Clinical and Laboratory Standards Institute, 2012), using Mueller Hinton broth II (MHB) (Becton Dickinson & Co., Sparks, MD, United States). MIC results were interpreted according to the European Committee on Antimicrobial Susceptibility Testing (http://www.eucast.org/clinical_breakpoints/) breakpoints for all antibiotics (European Committee on Antimicrobial Susceptibility Testing, 2018), but rifampicin, for which the French Society for Microbiology breakpoint was used (Soussy, 2012). Pentamidine has not susceptibility breakpoints defined.

The MIC value was the lowest concentration of antimicrobials that completely inhibited the bacterial growth. To determine the MBC values, 5-μL aliquots from the wells with no visible growth were spread on agar plates. The MBC value was the lowest concentration at which no colony formation occurred. Heteroresistance in the studied strains was also evaluated by reading the MIC at 24 and 48 h of incubation. Heteroresistance was defined when a fraction of the inoculum was able to grow two dilutions above the MIC value previously determined (Ferreira et al., 2015). All assays were performed in triplicates to ensure reproducibility.

### Time-kill curves

The concentrations used for pentamidine and the different antimicrobials tested corresponded to the MIC value obtained by microdilution. Moreover, with the Kp28 OXA-48/CTX-M-15 strain the assay was also performed at 1/2xMIC of pentamidine. Experiments were carried out with a starting inoculum of 5 × 10^5^ cfu/mL and the drugs alone or in combination. Tubes were incubated at 37°C, with shaking, and samples were taken at 0, 2, 4, 8, and 24 h, serially diluted and plated (Pournaras et al., 2011; Souli et al., 2011). Bactericidal activities of single drugs or combination were defined as a decrease ≥ 3 log_10_ cfu/mL from the starting inoculum, bacteriostatic effect was defined as no change respect to the initial bacterial concentration during the 24 h. Synergy was defined as a decrease ≥ 2 log_10_ cfu/mL for the drugs combination compared with the most active single agent (Pachón-Ibáñez et al., 2018). Experiments were performed three times on separate occasions.

### *In vitro* selection of resistant mutants

Time–kill curves were used. Strains elected strains were incubated with drugs at concentrations 1 × MIC, and Kp28 OXA-48/CTX-M-15 also at pentamidine concentration of 1/2 × MIC. Furthermore, the possible combinations of pentamidine plus the different studied antimicrobials were tested. Tubes with 20 mL of MHB with an inoculum of 5 × 10^5^ cfu/mL of each one of the strains were used. Tubes with the bacterial inoculum and without drugs were used as growth controls. The bacterial growth was counted at 0 and 24 h after incubation at 37°C. Ten-fold dilutions were made and 100 μL was plated on sheep blood agar and incubated for 24 h at 37°C. For the detection of resistant mutants, the MIC of each one of the studied drugs was carried out in triplicate for a maximum of five colonies at each time-point.

## Results

### MIC/MBC and heteroresistance

Individual MIC/MBC of each drug tested for the different clinical strains are shown in Table 1. All the strains were multidrug-resistant (MDR) (Magiorakos et al., 2012) except Kp28 OXA-48/CTX-M-15 which was resistant to rifampicin and fosfomycin. Heteroresistance was observed with tigecycline for the Kp1 NDM-1 (1 mg/L) and Kp21 VIM-1/DHA-1 strains (1 mg/L) and with doripenem for *E. cloacae* 32 (1 mg/L). The antibiotic susceptibility profiles are included in the Supplementary material.

**Table 1:** MIC/MBC of the different drugs for the eight carbapenemase-producing and/or colistin-resistant *Enterobacteriaceae* clinical strains.

**Clinical strains**	**MIC/MBC (mg/L)**						
	**PEN**	**GEN**	**AMK**	**TOB**	**RIF**	**TGC**	**DOR**
Kp07 VIM-1	400	4/16	1/1	4/4	32/32	0.5/1	1/2
Kp21 VIM-1/DHA-1	400	2/2	2/4	8 /16	> 256/> 256	0.25/> 4	> 4/> 4
Kp28 OXA-48 /CTX-M-15	400	0.25/0.25	1/1	0.5/0.5	16/16	1/4	0.5/0.5
Kp29 KPC-3	800	2/2	64/64	0.25/0.25	32/64	1/>8	> 4 / > 4
Kp1 NDM-1	400	>32/>32	> 128/> 128	> 32/> 32	> 256/> 256	0.25/> 4	1/2
Ec271 NDM-1	200	>32/>32	> 128/> 128	> 32/> 32	> 256/> 256	1/1	> 4/> 4
*E. cloacae* 32	800	8/16	2/4	4/8	8/8	0.5/> 4	0.25/> 4
*E. cloacae* 297	400	0.5/4	0.5/1	8/8	8/256	2/> 8	0.25/0.25

PEN, pentamidine; GEN, gentamicin; AMK, amikacin; TOB, tobramycin; TGC, tigecycline; RIF, rifampicin; DOR, doripenem; * Pentamidine breakpoints are not defined; GEN and TOB: * Susceptible, MIC ≤ 2 mg/L and resistant MIC > 4 mg/L; AMK, Susceptible, MIC ≤ 8 mg/L and resistant MIC > 16 mg/L; TGC and DOR; Susceptible, MIC ≤ 1 mg/L and resistant MIC > 2 mg/L and RIF: and resistant MIC > 16 mg/L.

### Time-kill curves

The bactericidal activity of the drugs alone is shown in Table 2. Pentamidine alone at 1xMIC was bactericidal from 2 to 24 h against six of the strains and from 4 to 24 h against Kp21 VIM-1/DHA-1; however, pentamidine alone was no bactericidal against *E. cloacae* 32. Doripenem showed bactericidal activity against three strains, and gentamycin, tobramycin, and amikacin were bactericidal against two strains each. Tigecycline alone was the only antimicrobial that showed not bactericidal effect against any strain.

**Table 2:** Bactericidal activity of drugs alone against eight of carbapenemase-producing and/or colistin-resistant *Enterobacteriaceae* clinical strains.

**Clinical strains**	**PEN**	**GEN**	**AMK**	**TOB**	**RIF**	**TGC**	**DOR**
Kp07 VIM-1	B (2–24 h)	–	B (4–8 h)	–	–	–	B (4 h)
Kp21 VIM-1/DHA-1	B (4–24 h)	B (4–24 h)	–	–	–	–	–
Kp28 OXA-48 /CTX-M-15	B (2–24 h)	–	–	–	–	–	–
Kp29 KPC-3	B (2–24 h)	B (2–24 h)	B (4–8 h)	B (2–8 h)	–	–	–
Kp1 NDM-1	B (2–24 h)	–	–	–	–	–	–
Ec271 NDM-1	B (2–24 h)	–	–	–	–	–	–
*E. cloacae* 32	–	B (24 h)	–	–	–	–	B (8–24 h)
*E. cloacae* 297	B (4–24 h)	–	–	B (8h)	–	–	B (8 h)

*PEN, pentamidine; GEN, gentamicin; AMK, amikacin; TOB, tobramycin; TGC, tigecycline; RIF, rifampicin; DOR, doripenem; B, Bactericidal; –: no bactericidal activity found; (): time frame in hours of the in vitro activity found*.

The *in vitro* activity of pentamidine in combination with antimicrobials is shown in Table 3. The bactericidal activity of pentamidine alone was also observed in combination. The combinations of pentamidine were synergistic against *E. cloacae 32* with amikacin, tobramycin and rifampicin at 24 h. Pentamidine plus rifampicin was the combination that showed synergistic activity against more strains: Kp21 VIM-1/DHA-1, Kp29 KPC-3 and *E. cloacae* 297 at 2 h and *E. cloacae* 32 at 24 h. Pentamidine plus doripenem did not show synergy against any strain.

**Table 3:** *In vitro* activity of pentamidine in combination with antimicrobials against eight of carbapenemase-producing and/or colistin-resistant *Enterobacteriaceae* clinical strains.

**Clinical strains**	**PEN + GEN**	**PEN + AMK**	**PEN + TOB**	**PEN + TGC**	**PEN + RIF**	**PEN + DOR**
Kp07 VIM-1	B (2–24 h)	B (2–24 h)	B (4–24 h)	B (2–24 h)	B (2–24 h)	B (2–24 h)
Kp21 VIM-1/DHA-1	B (2–24 h)	B (8–24 h)	B (8–24 h)	B (4–24 h)	B + S (2–24 h) + (2 h)	B (2–24 h)
Kp28 OXA-48/CTX-M-15	B (2–24 h)	B (2–24 h)	B (2–24 h)	B (2–24 h)	B (2–24 h)	B (2–24 h)
Kp29 KPC-3	B (2–24 h)	B (2–24 h)	B (2–24 h)	B (2–24 h)	B + S (2–24 h) + (2 h)	B (2–24 h)
Kp1 NDM-1	B (2–24 h)	B (2–24 h)	B (2–24 h)	B (2–24 h)	B (2–24 h)	B (2–24 h)
Ec271 NDM-1	B (4–24 h)	B (4–24 h)	B (4–24 h)	B (2–24 h)	B (2–24 h)	B (2–24 h)
*E. cloacae* 32	B (24 h)	B + S (8–24 h) + (24 h)	S (24 h)	–	B + S (24 h) + (24 h)	B (8–24 h)
*E. cloacae* 297	B (4–24 h)	B (2–24 h)	B (4–24 h)	B (4–24 h)	B + S (2–24 h) + (2 h)	B (4–24 h)

*PEN, pentamidine; GEN, gentamicin; AMK, amikacin; TOB, tobramycin; TGC, tigecycline; RIF, rifampicin; DOR, doripenem; B, bactericidal; S, synergistic; –: no in vitro activity found; (): time frame in hours of the in vitro activity found*.

**Figure 1:**
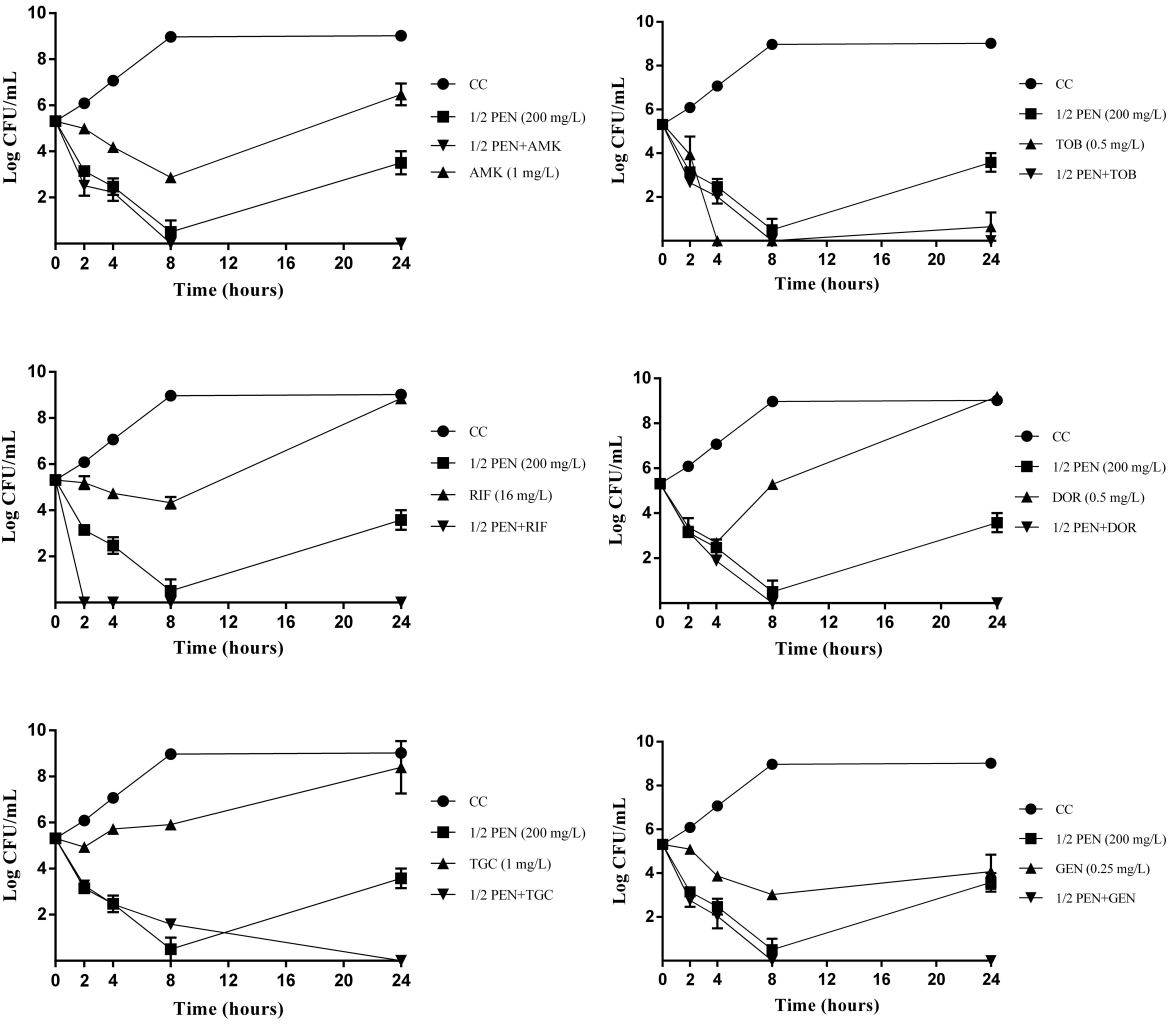
Time-kill curves for pentamidine (PEN) at 1/2xMIC in combination with antimicrobials against the clinical strain Kp28 OXA-48/CTX-M-15. CC: growth control, filled circles; PEN (1/2xMIC), filled squares; antimicrobials (1xMIC), filled triangles, combination of PEN + antimicrobial, filled inverted triangles.

The activity of pentamidine at 1/2xMIC in combination with antimicrobials against Kp28 OXA-48/CTX-M-15 is showed in Figure 1. Pentamidine 1/2xMIC plus antimicrobials showed synergism at 24 h, but the combination with tobramycin due to the high bactericidal activity of tobramycin at 24 h.

### *In vitro* selection of resistant mutants

The high bactericidal activity of pentamidine in combination with antimicrobials, achieving bacterial concentrations close to 0 log cfu/mL did not allow to analyze the selection of resistant mutants after 24 h incubation.

## Discussion

This study evaluates the use of pentamidine as antimicrobial agent against clinical strains of carbapenemase-producing and/or colistin-resistant Enterobacteriaceae, finding a strong *in vitro* activity both with pentamidine alone and combined with other antimicrobials, as aminoglycosides, tigecycline, doripenem, and rifampicin. Additionally, pentamidine was synergistic against selected strains in combination with some of these antimicrobials, especially when was studied combined with rifampicin.

Pentamidine showed a MIC rage against eight clinical strains of carbapenemase-producing and/or colistin-resistant Enterobacteriaceae from 200 to 800 mg/L. Due to its use as an antiprotozoal agent (Nguewa et al., 2005), no susceptibility breakpoints for pentamidine are defined. However, the MIC values obtained are in accordance to those reported analyzing the *in vitro* activity of pentamidine and five pentamidine analogs against a *E. coli* strain, with MIC values ranging from 100 to > 200 mg/L (Stokes et al., 2017). Moreover, we found that pentamidine at MIC concentration is bactericidal against seven of the eight tested strains; furthermore, at 1/2xMIC was bactericidal against the Kp28 OXA-48/CTX-M-15 producer strain.

Besides the robust bactericidal effect found with pentamidine alone, more important is that its combinations with the different antimicrobials tested potentiates the effect of these antimicrobials alone against the clinical strains using the time-kill assay. The *in vitro* activity observed with pentamidine in combination with antibiotics suggests there is strong possibility to repurpose it for antibacterial use against these difficult to treat MDR GNB (Pachón-Ibáñez et al., 2018; Rodríguez-Baño et al., 2018). These results are in accordance to the ones reported by Stokes et al. in which they found that the combination of pentamidine with rifampicin, novobiocin, erythromycin, or vancomycin potentiated the antimicrobials alone against a wild-type *E. coli* strain using chequerboard broth microdilution assays (Stokes et al., 2017). It is noteworthy that synergistic activity was observed when pentamidine was combined with amikacin, tobramycin, tigecycline, and/or rifampicin against the colistin-resistant *E. cloacae* 32, strain against which pentamidine alone did not show bactericidal activity. We would also like to mention, that no more synergistic effect with pentamidine in combination was observed due to the excellent bactericidal activity found with pentamidine alone at MIC concentration.

Pentamidine plus rifampicin was the combination that showed synergism against more of the tested strains (five out of eight). This excellent activity was also pointed out in the Stokes et al. study, in which pentamidine synergized with rifampicin against a wide phylogenetic distribution of antibiotic-resistant strains, including naturally polymyxin-resistant *Serratia* species (Stokes et al., 2017). The combination of rifampicin with other antimicrobials has been proved to be useful, both *in vitro* and *in vivo*, against other MDR GNB as *Acinetobacter baumannii* (Pachón-Ibáñez et al., 2010), as other example of repurposing a drug, such as rifampicin, previously used in staphylococcal and mycobacterial infections.

In summary, these results suggest that pentamidine, alone or in combination, may be a new alternative for the treatment of infections caused by carbapenemase-producing and/or colistin-resistant Enterobacteriaceae. To investigate further the possible usefulness of pentamidine new data from pharmacokinetics and pharmacodynamics and *in vivo* efficacy in experimental models of infection, including the dosage and safety, are required.

## Author contributions

MP-I has planned and coordinated the experiments, analyzed the results, and written the manuscript. GL-H and TC-C had performed the *in vitro* experiments. YS had reviewed the manuscript. RÁ-M, EC-M, and JP had reviewed the manuscript and the experiments.

### Conflict of interest statement

The authors declare that the research was conducted in the absence of any commercial or financial relationships that could be construed as a potential conflict of interest.
