# Supplementary material for: In vitro Activity of Pentamidine Alone and in Combination With Aminoglycosides, Tigecycline, Rifampicin, and Doripenem Against Clinical Strains of Carbapenemase-Producing and/or Colistin-Resistant Enterobacteriaceae
Source: Front Cell Infect Microbiol. 2018 Oct 18;8:363. doi: 10.3389/fcimb.2018.00363 (PMC6201057; doi:10.3389/fcimb.2018.00363)
Supplement: Supplementary file 1 [file Data_Sheet_1.PDF]

TABLE 1. MIC / MBC of the different drugs for the eight carbapenemase-producing and/or colistin-resistant *Enterobacteriaceae* clinical strains.

**Clinical strains**	**MIC (mg/L) / MBC (mg/L) (SR)***								
	**COL**	**GEN**	**AMK**	**TOB**	**RIF**	**TGC**	**DOR**	**MER**	**FOF**
**Kp07 VIM-1**	0.25 (S) / 0.5	4 (R) / 16	1 (S) / 1	4 (R) / 4	32 (R) / 32	0.5 (S) / 1	1 (S) / 2	1(S) / 1	>64 (R) / >64
**Kp21 VIM-1/DHA-1**	>32 (R) / >32	2 (S)/ 2	2 (S) / 4	8 (R) / 16	>256 (R) / >256	0.25 (S) / >4	>4 (R) / >4	>16 (R) / >16	>64 (R) / >64
**Kp28 OXA-48/CTX-M-15**	0.5 (S) / 1	0.25 (S) / 0.25	1 (S) / 1	0.5 (S) / 0.5	16 (R) / 16	1 (S) / 4	0.5 (S) / 0.5	1(S)/ 1	>64 (R) / >64
**Kp29 KPC-3**	16 (R) / 32	2 (S) / 2	64 (R) / 64	0.25 (S) / 0.25	32 (R) / 64	1 (S) / >8	>4 (R) / >4	>16 (R) / >16	>64 (R) / >64
**Kp1 NDM-1**	0.5 (S) / 1	>32 (R) / >32	>128 (R) / >128	>32 (R) / >32	>256 (R) / >256	0.25 (S) / >4	1 (S) / 2	>16 (R) / >16	>64 (R) / >64
**Ec271 NDM-1**	0.5 (S) / 1	>32 (R) / >32	>128 (R) / >128	>32 (R) / >32	>256 (R) / >256	1 (S) / 1	>4 (R) / >4	>16 (R) / >16	>64 (R) / >64
***E. cloacae* 32**	>512 (R) / >512	8 (R) / 16	2 (S) / 4	4 (R) / 8	8 (S) / 8	0.5 (S) / >4	0.25 (S) / >4	0.5 (S) / 0.5	>64 (R) / >64
***E. cloacae* 297**	8 (R) / 16	0.5 (S) / 4	0.5 (S) / 1	8 (R) / 8	8 (S) / 256	2 (R) / >8	0.25 (S) / 0.25	0.125 (S) / 0.125	>64 (R) / >64

COL: colistin; GEN: gentamicin; AMK: amikacin; TOB: tobramycin; RIF: rifampicin; TGC: tigecycline; DOR: doripenem; MER: Meropenem; FOF: Fosfomycin. Breakpoints, COL: Susceptible, MIC ≤ 2 mg/L and resistant MIC > 2 mg/L; GEN and TOB: * Susceptible, MIC ≤ 2 mg/L and resistant MIC > 4 mg/L; AMK: Susceptible, MIC ≤ 8 mg/L and resistant MIC > 16 mg/L; TGC and DOR; Susceptible, MIC ≤ 1 mg/L and resistant MIC > 2 mg/L and RIF: and resistant MIC > 16 mg/L; MER: Susceptible, MIC ≤ 2 mg/L and resistant MIC > 8 mg/L; FOF: Susceptible, MIC ≤ 32 mg/L and resistant MIC > 32 mg/L; * S: Susceptible and R: Resistant
